# Supplementary material for: Social and emotional contexts predict the development of gaze following in early infancy
Source: R Soc Open Sci. 2020 Sep 16;7(9):201178. doi: 10.1098/rsos.201178 (PMC7540771; doi:10.1098/rsos.201178)
Supplement: Supplementary Information [file rsos201178supp1.docx]

Supplementary

Social and emotional contexts predict the development of gaze following in early infancy

Kim Astor, Marcus Lindskog, Linda Forssman, Ben Kenward, Mari Fransson, Alkistis Skalkidou, Anne Tharner, Juliëtte Cassé, & Gustaf Gredebäck

**Methods**

**Participants**

The sample consisted of the participants in the BASICchild cohort, a longitudinal project that aimed to follow 120 infants from 6 to 30 months of age. Families were originally recruited at gestational week 32 from an epidemiological project on maternal health and pregnancy (the BASIC project, see Iliadis et al., 2015; Wesström, Skalkidou, Manconi, Fulda, & Sundström-Poromaa, 2014). This was done using a questionnaire supplemented with information about the project. Applied exclusion criteria was significant physical health problems (both mother and infant) and preterm birth (< 36 weeks gestation). In order to assess the impact of maternal depression on early development, all mothers with major clinical depression were invited (A and C criteria according to the DSM-IV guidelines, assessed with the Depression Self Rating Scale; Svanborg & Ekselius, 2003). Further, since the study was conducted in a university city where the educational level is relatively high, we applied stratified sampling. Thus, mothers with lower educations were over-selected. In the final sample approximately 28% of the mothers reported low education (≤ 12 years education) which is representative of the 29% in the general Swedish population (SEB, 2016).

**Measures and procedures**

**Maternal postpartum depression.** We used the Edinburgh Postnatal Depression Scale (EPDS; Cox, Holden, & Sagovsky, 1987) to assess maternal depressive symptoms at 6 weeks, 6 months, and 12 months after child delivery. The EPDS is a self-report 10-item inventory that measures depressive symptoms during the past seven days. The instrument was initially developed to screen for depression in the first 6-8 weeks postpartum, but it has also shown to be a valid measure for assessing mothers’ depressive symptoms beyond the first postnatal year (Cox, Chapman, Murray, & Jones, 1996). Each item is rated on a 4-point scale from 1 to 4 (for example, “not at all” to “yes most of the time”). In the current sample, the EPDS scale had excellent reliability at the 6 weeks, 6 months, and 12 months postpartum assessments (Cronbach’s alpha = .90, and .89 and .89, respectively). The mean scores from the EPDS, ranging from 1 to 4, were used to index depressive symptoms at the three assessment points (6 weeks, 6 months and 12 months), with high values indicating higher levels of depressive symptoms.

| Table S1 |  | | | |  |  |
| --- | --- | --- | --- | --- | --- | --- |
| *Descriptive statistics for the Edinburgh Postnatal Depression Scale* | | | | | | |
| Age of infant | | N | *Min* | *Max* | *M* | *SD* |
| 6 weeks | | 109 | 1.00 | 3.40 | 1.67 | .50 |
| 6 months | | 117 | 1.00 | 3.70 | 1.81 | .58 |
| 12 months | | 93 | 1.00 | 3.70 | 1.57 | .52 |
|  |  | | | |  |  |

**Infant-mother attachment.** The Strange Situation Procedure (SSP; Ainsworth et al., 1978) was used to assess infant-mother attachment quality when the child was 12 months old. The SSP contains brief episodes of increasing stress for the infants, including two infant-mother separations and reunions. In short, the infant is introduced to an unfamiliar laboratory environment and a female stranger. The mother leaves the room and then returns to the room twice. During the first infant-mother separation the infant is alone with the stranger and during the second separation the infant is alone by him/herself. Infants’ attachment behavior during the SSP was coded from videotapes, according to Ainsworth et al. (1978) and Main and Solomon’s (1990) coding systems, by two certified coders. The two coders were blind to all other participant information and to specific study aims. Half (*n* = 60) of the SSPs episodes were coded by both coders. Infants’ proximity-seeking, contact-maintenance, contact-resistance and proximity-avoidance, based on the two reunion episodes, was rated on a 7-point scale in order to classify the infants as secure (B), insecure-avoidant (A), or insecure-resistant (C). Then, disorganized (D) behaviors were rated during all episodes when the mother was present on a 1 to 9-point scale with scores above 5 leading to a D classification. In the full sample of 112 infants assessed with the SSP the distribution was B = 63 (53 %), A = 9 (8 %), C = 13 (11 %) and D = 27 (23 %). On the basis of the ABCD classification we used the B (secure) vs ACD (non-secure) classifications. This is because we are not primarily interested in the different dimensions of attachment but rather to use it as a proxy for infant’s social and emotional context, targeting secure and non-secure groups. Interrater reliability using the four-way classification system (B vs ACD) was moderate (*kappa* = .58). Disagreements on the double-coded reliability set were resolved by discussion by the two coders.

**Analyses**

**Path model script and data**

The eye-tracking data analyses were conducted in TimeStudio and the workflow is openly available in the Dryad repository (Astor, 2020). To get access to the eye-tracking raw data and the other variables, please contact the corresponding author. Below is the R script for the path analysis. To run the script you need the R statistical software, available at <https://www.r-project.org/>.

library(lavaan)

library(influence.SEM)

GF_data <- read.csv2('BASIC_GF.csv')

Model <- '

GF_10 ~ GF_6 + Dep12mpp + Dep6mpp + Dep6vpp + BvsACD

GF_6 ~ Dep6mpp + Dep6vpp + BvsACD

Dep12mpp ~ Dep6mpp + BvsACD

Dep6mpp ~ Dep6vpp + BvsACD

Dep6vpp ~ BvsACD

'

gCD.mod<- genCookDist(Model,data=GF_data,std.lv=TRUE)

plot(gCD.mod,pch=19,xlab="observations",ylab="Cook distance")

GF_data.gCDExc <- GF_data[-which(gCD.mod>1),]

Model.fit <- sem(Model, data = GF_data.gCDExc, estimator = 'ML', missing='fiml', std.ov = TRUE)

summary(Model.fit, fit.measures = TRUE,standardize = TRUE, rsquare = TRUE)

| Table S2 | | |
| --- | --- | --- |
| *Path coefficients and associated p values* | | |
|  | β | *p* |
| Attachment → Gaze Following 6 months | .413 | .034 |
| Attachment → Gaze Following 10 months | .349 | .073 |
| Attachment → Postpartum depression at 6 weeks | -.208 | .280 |
| Attachment → Postpartum depression at 6 months | .179 | .245 |
| Attachment → Postpartum depression at 12 months | -.089 | .580 |
| Postpartum depression 6 weeks → Postpartum depression at 6 months | .665 | <.001 |
| Postpartum depression 6 weeks → Gaze Following 6 months | -.092 | .466 |
| Postpartum depression 6 weeks → Gaze Following 10 months | .214 | .099 |
| Postpartum depression at 6 months → Postpartum depression at 12 months | .608 | <.001 |
| Postpartum depression at 6 months → Gaze Following 6 months | .117 | .355 |
| Postpartum depression at 6 months → Gaze Following 10 months | -.176 | .216 |
| Postpartum depression at 12 months → Gaze Following 10 months | -.364 | .013 |
| Gaze Following 6 months → Gaze Following 10 months | .130 | .181 |
|  | | |

References

Ainsworth, M. D. S., Blehar, M. C., Waters, E., & Wall, S. (1978). Patterns of attachment: A psychological study of the strange situation. Oxford: Lawrence Erlbaum.

Astor K. 2020 Data from: Social and emotional contexts predict the development of gaze following in early infancy. Dryad Digital Repository. (doi:10.5061/dryad.v41ns1rs5)

Cox, J. L., Chapman, G., Murray, D., & Jones, P. (1996). Validation of the Edinburgh Postnatal Depression Scale (EPDS) in non-postnatal women. Journal of Affective Disorders, 39(3), 185-189. <https://doi.org/10.1016/0165-0327(96)00008-0>

Cox, J., Holden, J., & Sagovsky, R. (1987). Detection of postnatal depression. development of the 10-item edinburgh postnatal depression scale. *The British Journal of Psychiatry, 150*(6), 782-786. doi:10.1192/bjp.150.6.782

Iliadis, S. I., Koulouris, P., Gingnell, M., Sylvén, S. M., Sundström-Poromaa, I., Ekselius, L., ... & Skalkidou, A. (2015). Personality and risk for postpartum depressive symptoms. *Archives of Women's Mental Health, 18*(3), 539-546. <https://doi.org/10.1007/s00737-014-0478-8>

Main, M., & Solomon, J. (1990). Procedures for identifying infants as disorganized/disoriented during the Ainsworth Strange Situation. *Attachment in the preschool years: Theory, research, and intervention*, *1*, 121-160.

Statistiska Centralbyrån (2018-11-06). Utbildningsnivån i Svergie. [Electronic]. Stockholm. Available: https://hv.se.libguides.com/c.php?g=243055&p=1617016 [2018-11-06].

Svanborg, P., & Ekselius, L. (2003). Self-assessment of DSM-IV criteria for major depression in psychiatric out- and inpatients. *Nordic Journal of Psychiatry, 57*(4), 291-296. doi:10.1080/08039480310002084
